# Gray's Supplement to the Pharmacopœia, &c.

**Published:** 1847-07-01

**Authors:** 


Gray's Supplement to the Pharmacopœia; being a concise but comprehensive Dispensatory and Manual of Facts and Formulæ. Entirely re-written, re-arranged, and considerably enlarged. By *Theophilus Redwood,* Professor of Pharmacy to the Pharmaceutical Society of Great Britain. Octavo, pp. 1118. London, 1847.

Mr. Redwood has added a great deal of interesting and very useful information to this new edition of a well-known work. The introductory chapters, containing a chronological history of Pharmacopœias and Dispensatories, an account of weights and measures, description of the methods of taking specific gravities, tables of thermometrical equivalents, &c., a pharmaceutical calendar, and various other matters, have been carefully and correctly prepared. We observe some omissions in the more strictly pharmaceutic department, that Mr. R. will do well to supply in his next edition. For example, we happened to look into the work for the composition of Ruspini's styptic, but found no notice of it. Such well-known formulæ, too, as *vinum ferri, vinum antimonii,* ought surely to have had a place. The addition of some toxicological tables also would be of service. To find room for these and various other matters unnoticed, much of the chapter on Animals might be left out without any detriment to the usefulness of the work.

